# Healthcare-associated bacterial infections in the paediatric ICU

**DOI:** 10.1093/jacamr/dlaa066

**Published:** 2020-09-14

**Authors:** Olugbenga Akinkugbe, Fiona J Cooke, Nazima Pathan

**Affiliations:** 1 Paediatric Intensive Care Unit, Cambridge University Hospitals NHS Foundation Trust, Cambridge, UK; 2 Clinical Microbiology and Public Health Laboratory, National Infection Service, PHE, Cambridge University Hospitals NHS Foundation Trust, Cambridge, UK; 3 Girton College, University of Cambridge, Cambridge, UK; 4 King’s College, University of Cambridge, Cambridge, UK

## Abstract

**Background:**

An estimated 3.2 million patients annually develop healthcare-associated infections (HCAIs) in Europe alone amid the major challenge of increasing antimicrobial resistance. Critically ill children warrant specific evaluation because of differences in epidemiology, causative organisms and infection sites.

**Objectives:**

To examine the prevalence and antimicrobial susceptibility patterns of three types of HCAI in critically ill children and determine the effect on their disease course.

**Materials and methods:**

Retrospective cohort review of critically ill children admitted to a general paediatric ICU (PICU) at a regional academic tertiary referral centre over a 3 year period.

**Results:**

There were 1930 admissions with a median age of 38 months. Children with HCAIs had a higher incidence of comorbidities (74% versus 24%) and a longer median length of stay (8 days versus 3 days). We identified 26 positive isolates (blood, lower respiratory and urine) taken 48 h or more after admission. The combined incidence was 1.34%. Hospital-acquired pneumonia accounted for 58% of HCAIs, urinary tract infections for 31% and bloodstream infections for 11%. The majority (61.5%) of HCAIs were caused by Gram-negative organisms. Seven isolates were resistant to antimicrobials used to treat HCAI. All of these were Gram-negative organisms (*Pseudomonas aeruginosa*, *Klebsiella oxytoca* and *Escherichia coli*).

**Conclusions:**

These data revealed a low incidence of HCAIs, 27% of which were resistant Gram-negative organisms. Critically ill children with HCAIs were more likely to have comorbidities and an increased length of stay. These factors may increasingly impact on PICU bed availability, an already limited resource.

## Introduction

Healthcare-associated infections (HCAIs) are some of the most frequent adverse outcomes in healthcare delivery. A recent point prevalence study involving 29 EU member states found a 6% prevalence of HCAI and estimated that 3.2 million patients develop an HCAI each year.^1^ Global data on HCAI are limited because of the wide variability of surveillance, diagnostic criteria, reliability of data collection and reporting across the world. However, it is well recognized that patients acquiring HCAIs are at increased risk of adverse outcomes including prolonged hospital stay, mortality risk and increased antimicrobial resistance (AMR). In addition, HCAI and AMR are also associated with a massive financial burden.^2^ The financial loss associated with directs costs is estimated at €7 billion each year in Europe. In the USA the annual economic impact was estimated at US$6.5 billion in 2004.^2^

AMR represents one of the greatest challenges in healthcare and it is set to increase. From a historical perspective, antibiotic resistance was observed in bacteria soon after antibiotics were introduced as treatment for infections.^3^ As antibiotic use has increased, so have the level and complexity of resistance mechanisms developed by bacteria.^4^

There is increasing recognition that HCAIs are a significant problem in all groups of hospitalized patients. The most vulnerable are the sickest patients and those at extremes of age. International studies have highlighted differences between children and adults in terms of epidemiology, causative organisms and infection sites. However, there are few data regarding the critically ill paediatric population, where antimicrobial use is widespread.^5^ The few studies in this area have shown that the spectrum of HCAI identified in paediatric ICUs (PICUs) differs from other inpatient paediatric settings and warrants specific evaluation.

A recent point prevalence study showed that the incidence of HCAI was higher in PICUs than in any other paediatric department. Furthermore, there was a significant amount of AMR in both Gram-positive and Gram-negative bacterial isolates.^6^

Within this context, increasing numbers of children with life-limiting conditions are being admitted to PICUs and now account for the majority of bed days on PICUs. Children in this population have complex care needs and frequently attend a variety of healthcare settings.^7^

PHE recently reported that the greatest threat of AMR is from Gram-negative bacteria, with *Escherichia coli*, *Klebsiella pneumoniae* and *Pseudomonas aeruginosa* accounting for 72% of all Gram-negative bloodstream infections in the UK.^8^ Resistant strains of staphylococci and enterococci indicate that Gram-positive organisms are also of significant concern.^4^

Resistance may result from *de novo* mutation of genes or horizontal transfer of genetic elements from other organisms carrying resistance genes leading to a variety of enzymatic- and non-enzymatic-mediated pathways.^9^

Enzyme-mediated resistance includes expression of enzymes that destroy antimicrobial drugs, efflux systems that prevent drugs from reaching intracellular target sites, modification of target sites, or alternative metabolic pathways that bypass the action of antimicrobials.^4^ The effectiveness of resistance mechanisms is demonstrated by the rapid spread of resistant strains. In addition to the rise in ESBL-producing Enterobacteriaceae and AmpC-producing organisms, carbapenemase-producing strains have emerged as a result of enzymes such as Verona Integron-Mediated (VIM) and New Delhi-type MBLs (NDM), OXA-48 and *K. pneumoniae* carbapenemase (KPC). This diversity in mechanisms of AMR is a cause of major concern, particularly for patients in ICUs.^9^

In this study, we examined the prevalence and epidemiology of HCAI in critically ill children. To this end we surveyed the cultures of blood, urine and endotracheal secretions taken after 48 h of admission to the PICU to identify potential HCAI. We examined whether antimicrobial susceptibility patterns of the causative organisms affected the course of their disease.

## Methods

We carried out a retrospective review of children admitted to the PICU at a large regional academic tertiary referral centre over a 3 year period between November 2014 and October 2017.

We identified all patients admitted to the PICU and interrogated the electronic patient record to identify all patients with positive microbiological samples (blood, urine or endotracheal secretions) collected following admission to the PICU. We collected demographic information and reviewed each patient record to identify clinical, radiological and microbiological features (as relevant) that were consistent with bloodstream infection, urinary tract infection or lower respiratory tract infection.

Inclusion criteria were: (i) children and young people aged under 16 years admitted to the PICU at Addenbrooke’s Hospital between 1 November 2014 and 31 October 2017, inclusive; (ii) hospital admission of 48 h or more; and (iii) positive culture of blood, urine, sputum or bronchoalveolar lavage samples taken 48 h or more after admission and associated clinical features of bloodstream infection, urinary tract infection or lower respiratory tract infection, respectively.^10^

Exclusion criteria were: (i) bloodstream, urinary tract or lower respiratory tract infections that began before, or at the time of, admission; (ii) colonization with organisms without adverse clinical features; and (iii) children with clinical features of bloodstream infection, urinary tract infection, or lower respiratory tract infection but with negative culture of blood, urine and endotracheal secretion samples, respectively.

We excluded duplicate samples from the same hospital-acquired infection that showed identical results but included those from which different organisms were isolated.

**Figure 1.**
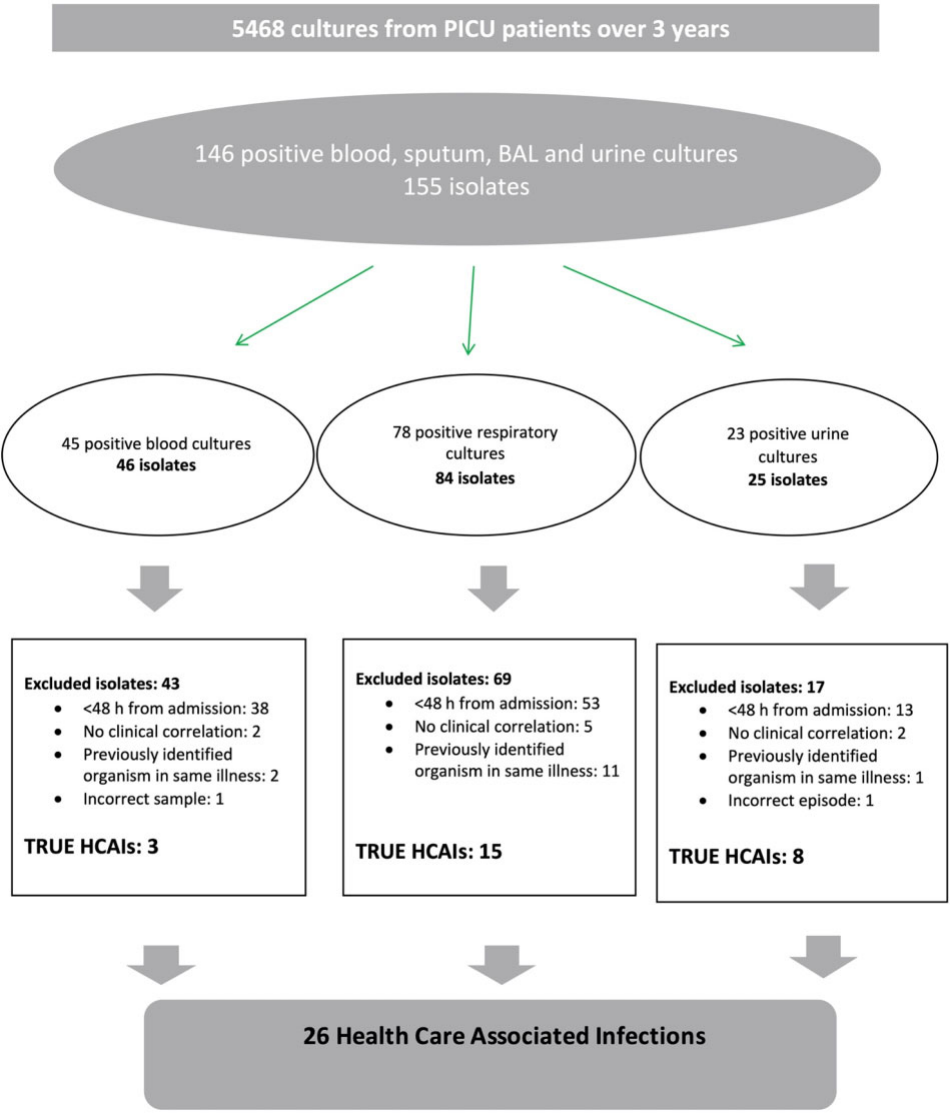
Flowsheet for identifying HCAIs. BAL, bronchoalveolar lavage.

Finally, we reviewed the susceptibility patterns of the organisms identified. At the time of the study, the empirical antibiotic treatment for suspected HCAI was either piperacillin/tazobactam and gentamicin in combination, or meropenem alone. Departmental guidelines recommended discussion with a microbiologist before selecting antimicrobials for HCAI in patients with allergy to penicillin or cephalosporins. We specifically sought to identify isolates resistant to these antibiotics. We also sought to identify the incidence of MRSA, ESBL-producing organisms, VRE and carbapenemase-producing Enterobacteriaceae. The process by which HCAIs were identified is illustrated in Figure 1.

Descriptive data are presented as numbers and percentages. Means and SD or medians and IQR are used as appropriate.

### Ethics

The study was categorized and approved as a service evaluation by our institution. Ethics committee approval and patient consent were not required for this retrospective study.

## Results

During the 3 year period studied there was a total of 1930 admissions to the PICU, of which 44% were female. Median age was 38 months (10–118). Demographic and diagnostic data are outlined in Table 1. Respiratory conditions accounted for the largest primary diagnostic group, followed by surgical conditions.

**Table 1.** Demographic and diagnostic data for all patients

Category	Value
Total PICU admissions November 2014–October 2017	1930
Female, *n* (%)	847 (44)
Age range	0 days–18 years 4 months
Median (IQR) age (months)	38 (10–118)
Admissions by primary diagnostic group, *n* (%)	
respiratory	661 (34.2)
surgical	396 (20.5)
oncology	226 (11.7)
neurology (excluding trauma)	206 (10.7)
trauma	148 (7.6)
infection	127 (6.6)
cardiovascular	50 (2.6)
gastrointestinal	29 (1.5)
endocrine/metabolic	29 (1.5)
blood/lymphatic (non-oncology)	20 (1)
musculoskeletal	7 (0.4)
other	31 (1.6)
Mechanical ventilation, *n* (%)	735 (38)
Median (IQR) length of mechanical ventilation (days)	4 (2–6)
Comorbidities in all patients, *n* (%)	470 (24.4)
Comorbidities in patients with HCAI, *n* (%)	17 (74)
Median (IQR) LOS for all patients (days)	3 (2–6)
Median (IQR) LOS for patients without HCAI (days)	3 (2–5.5)
Median (IQR) LOS for patients with HCAI (days)	8 (5.25–10)
Median (IQR) LOS for patients with resistant HCAI (days)	7.5 (4.5–10)
Mortality, *n* (%)	63 (3.3)

The cultures of blood, respiratory secretions and urine taken at least 48 h after PICU admission yielded a total of 26 positive isolates from 23 different patients. One patient had positive cultures from endotracheal secretions on three different admissions. In another patient two different organisms were isolated from urine culture during the same illness.

The combined incidence of the three HCAIs was 1.34%. The highest numbers of HCAIs were pneumonias (58%), followed by urinary tract infections (31%).

**Figure 2.**
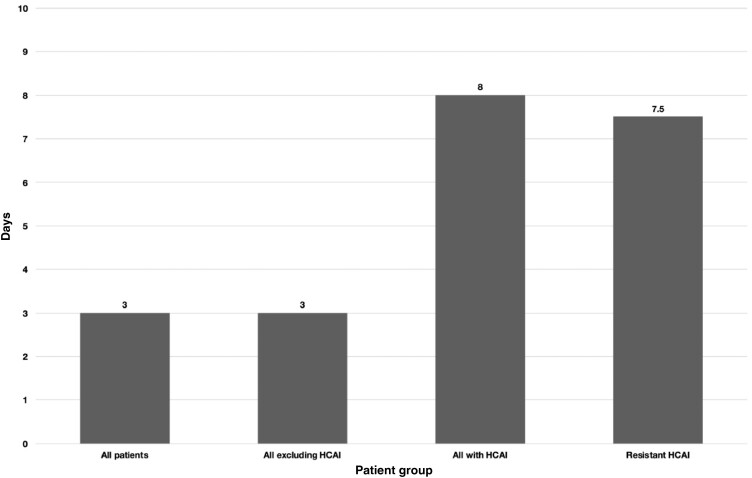
Median LOS for all groups.

Almost one quarter of all children admitted to the PICU had one or more comorbidities. Seventy-four percent of patients in whom an HCAI was identified had one or more significant comorbidities. Forty percent of patients with HCAI had documentation of a previous PICU or neonatal ICU (NICU) admission. The median length of stay (LOS) for all patients admitted to PICU within the study period was 3 days (2–6). The median LOS was 8 days (5.25–10) for patients with HCAI. These data are shown in Figure 2.

**Figure 3.**
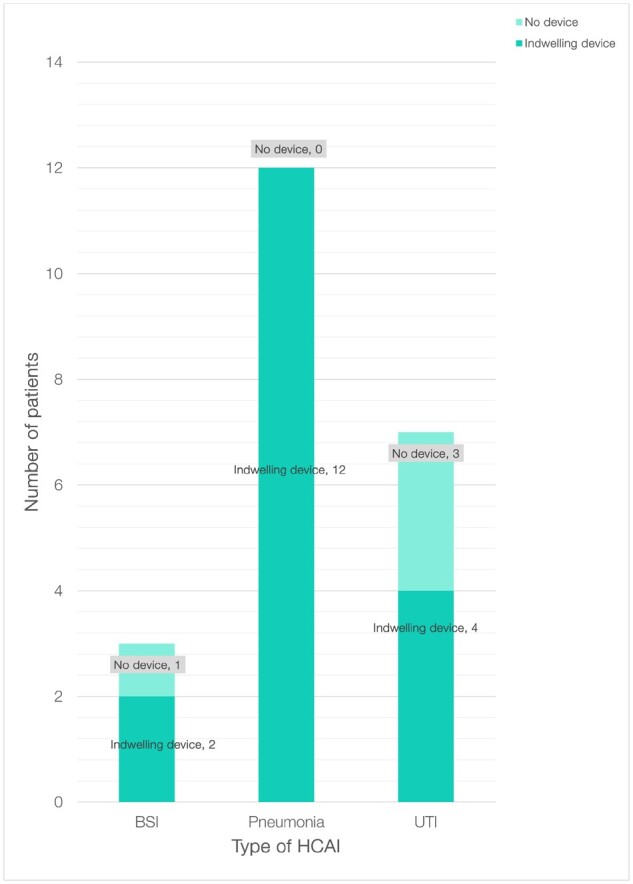
Indwelling devices in patients with HCAI. BSI, bloodstream infection; UTI, urinary tract infection.

All cases of hospital-acquired pneumonia (HAP) were associated with endotracheal tubes. The majority of bloodstream infections and urinary tract infections were associated with central venous and urinary catheters, respectively, as shown in Figure 3.

A broad distribution of organisms was identified in patients with HCAI, of which 16 (61.5%) were Gram-negative organisms and 10 (38.5%) were Gram-positive organisms. This is shown in Table 2.

**Table 2.** Organisms and AMR in patients with HCAI

Type of HCAI	Value, *n* (%)	
bloodstream infection	3 (11.5)	
pneumonia	15 (57.7)	
UTI	8 (30)	
Gram-positive organisms	10 (38.5)	
*E. faecalis*	5 (19.2)	
*E. faecium*	2 (7.7)	
*S. aureus*	2 (7.7)	
*S. epidermidis*	1 (3.8)	
Gram-negative organisms	16 (61.5)	
*P. aeruginosa*	5 (19.2)	
*E. coli*	4 (15.4)	
*K. oxytoca*	3 (11.5)	
*Haemophilus influenzae*	2 (7.7)	
*Citrobacter freundii*	1 (3.8)	
*K. pneumoniae*	1 (3.8)	

Type of HCAI	Organism	Resistance

bloodstream infection	*E. faecium*	nil
bloodstream infection	*S. epidermidis*	nil
bloodstream infection	*E. faecalis*	nil
pneumonia	*C. freundii*	nil
pneumonia	*E. faecium*	nil
pneumonia	*H. influenzae*	nil
pneumonia	*H. influenzae*	nil
pneumonia	*K. oxytoca*	nil
pneumonia	*K. oxytoca*	ceftazidime
pneumonia	*K. oxytoca*	piperacillin/ tazobactam
pneumonia	*K. pneumoniae* (ESBL)	nil
pneumonia	*P. aeruginosa*	ciprofloxacin
pneumonia	*P. aeruginosa*	ertapenem
pneumonia	*P. aeruginosa*	imipenem
pneumonia	*P. aeruginosa*	nil
pneumonia	*P. aeruginosa*	nil
pneumonia	*S. aureus*	nil
pneumonia	*S. aureus*	nil
UTI	*E. faecalis*	nil
UTI	*E. faecalis*	nil
UTI	*E. faecalis*	nil
UTI	*E. faecalis*	nil
UTI	*E. coli*	nil
UTI	*E. coli*	nil
UTI	*E. coli*	gentamicin
UTI	*E. coli*	gentamicin

UTI, urinary tract infection.

There was marked variation in the organisms identified in different types of HCAI. All three hospital-acquired bloodstream infections were with Gram-positive organisms [*Enterococcus faecalis* (1), *Enterococcus faecium* (1) and *Staphylococcus epidermidis* (1)]. Gram-negative organisms, particularly Enterobacteriaceae and *Pseudomonas* were dominant among the 15 patients with HAP. The eight urinary tract infections were due to *E. faecalis* (four isolates) and *E. coli* (four isolates).

Our review of the susceptibility pattern of the 26 hospital-associated isolates identified few organisms with significant resistance. Overall there were seven isolates with resistance to antimicrobials commonly used as treatment for HCAI. All were Gram-negative organisms: three *P. aeruginosa*, two *Klebsiella oxytoca* and two *E. coli*. Five of the seven resistant isolates were from sputum and two were obtained from urine. We looked specifically at resistance to piperacillin/tazobactam, ceftazidime, glycopeptides, aminoglycosides and carbapenems. In relation to *Pseudomonas* we also looked at resistance to ciprofloxacin.

Among the five endotracheal isolates with resistance, there was one *K. oxytoca* resistant to piperacillin/tazobactam (susceptible to ceftazidime) and one *K. oxytoca* resistant to ceftazidime (susceptible to piperacillin/tazobactam). Among the three endotracheal isolates with resistant *P. aeruginosa*, one was resistant to ciprofloxacin and two were resistant to carbapenems. Although an ESBL-producing *K. pneumoniae* was isolated from one endotracheal sample, both disc testing and determination of MIC demonstrated susceptibility to gentamicin and meropenem. *E. coli* resistant to gentamicin was isolated from two urine samples. Both isolates were susceptible to amikacin. These data are shown in Table 2.

There were no resistant organisms identified among hospital-acquired bloodstream infections. The three Gram-positive isolates from blood cultures were susceptible to either vancomycin or teicoplanin. There were no hospital-acquired infections with MRSA, VRE or carbapenemase-producing Enterobacteriaceae in our study.

## Discussion

### Incidence of HCAIs

The primary focus of this study was the incidence of healthcare-associated bloodstream, respiratory and urinary tract infections and the resistance patterns of their causative bacteria. Our data showed an incidence of HCAI of 1.34%. This is lower than reported in previous studies.

Richards *et al.*^5^ reported a mean overall infection rate of 6.1% in their prospective study of 61 PICUs published in 1999. Raymond *et al.*^11^ reported an overall incidence of 2.4% across all paediatric wards and an alarmingly high incidence of 23.6% across five PICUs in their European multi-centre prospective study published in 2000.

A recent point prevalence study of HCAIs in paediatric patients by the ECDC reported an overall prevalence of 4.2% across a variety of paediatric acute care facilities. They also found the highest prevalence in PICUs at 15.5%.^6^

It is important to note differences in methodology when interpreting these data. Positive microbiology was a requirement for inclusion in our study. Furthermore, our focus was on bloodstream infections, pneumonias and urinary tract infections, which accounted for the overwhelming majority of infections in the previous studies (80% in the 2000 study and over 70% in the 2017 study). Other studies included surgical site wound infections, gastrointestinal infections and viral and fungal infections, which were not included in our study. In light of the low incidence of HCAI, we suggest that inclusion of other infections would be unlikely to alter our results significantly. Furthermore, the diagnostic criteria in the earlier studies appear more liberal than the criteria we employed.

Despite the limitations described above, the low incidence we identified may be associated with greater recognition of the risks of HCAI and AMR. Although there are no baseline data for our PICU from previous years for comparison, successive years have seen implementation of infection control policies. These range from improvements in screening and surveillance, strict isolation protocols to prevent the spread of infection and formalized antimicrobial stewardship embedded through multidisciplinary rounds.

Despite these examples of good practice, there remains an ongoing need to reinforce and re-evaluate established preventative infection control measures such as hand hygiene, isolation, maintenance of asepsis during invasive procedures and timely removal of invasive devices.

### Comorbidities

Within the cohort of patients admitted to our PICU over the 3 year period, just under one quarter (24.4%) were noted to have at least one comorbidity. In contrast, over two-thirds (74%) of patients with HCAIs had one or more comorbidities. Children with comorbidities are more likely to have multiple admissions to PICUs, which in turn could result in increased antibiotic exposure, with implications for AMR. In the context of the increasing proportion of PICU admissions of children with complex and life-limiting conditions, this is a subject that requires further analysis.

### Distribution of HCAIs

In our study the distribution of HCAIs was different from previously reported studies. We found respiratory tract infections (57.7%) to be most common, followed by urinary tract infections (30.8%) then bloodstream infections (11.5%). Raymond *et al.*^11^ and Richards *et al.*^5^ reported that the most commonly identified HCAIs in PICUs were bloodstream infections followed by lower respiratory tract infections and urinary tract infections. Zingg *et al.*^6^ did not specifically describe the distribution of infections in PICUs but identified bloodstream infections as the most common, followed by lower respiratory tract infections, gastrointestinal infections, upper respiratory tract infections and urinary tract infections.^6^

The increased LOS in patients with HCAI (median of 8 days compared with 3 days) highlights the significant impact of HCAI on patient outcomes. Although the number of episodes of HCAI in our study was small (*n *=* *26), an increased LOS has a significant impact on costs and reduces PICU bed availability, the effect of which is more pronounced at times of seasonal high demand.

### Organisms and resistance

In our study, Gram-negative organisms accounted for the majority (61.5%) of all isolates and accounted for all of the organisms with resistance to commonly used antimicrobials. This reflects the expression of concern from PHE^8^ about the threat posed by Gram-negative organisms and alerts us to the potential for these bacteria to evolve mechanisms of resistance and potentially evade treatment with all current antimicrobials.^12^

As outlined earlier, although some bacteria have innate resistance to certain classes of antibiotics, of greater concern is the ability of initially susceptible bacteria to acquire resistance through mutations and selection pressures or by horizontal transfer of genetic material. These mechanisms have enabled organisms to develop MDR, a process that has been demonstrated *in vivo* with the demonstration of evolving resistance patterns of bacterial populations during courses of antibiotic treatment.^4^

Resistance to empirical antibiotics has been associated with increased mortality in patients with *Staphylococcus aureus*, CoNS, enterococci, *Enterobacter* spp., *P. aeruginosa*, *K. pneumoniae* and *E. coli*. Though we did not identify any resistant hospital-acquired bloodstream infections, the existence of resistant Gram-negative isolates from sputum and urine samples demands that we anticipate the emergence of such invasive infections.

### Wider context of HCAI and AMR in our institution

Our PICU is within a large regional academic centre with over 1000 inpatient beds. In the year 2016–17 the Infection Prevention and Control (IPC) indices ranked around the average for NHS acute trusts in England.^13^

Our findings on HCAI and AMR in the PICU should be considered within the wider context of HCAI and AMR at our institution. According to PHE AMR local indicators, cases of HCAI in our institution were generally lower than the average for England in 2017. Hospital-onset cases of *Klebsiella* spp., *P. aeruginosa* and MRSA were all within the lowest or second lowest quintile in England. However, cases of hospital-onset *E. coli* ranged from the middle to the highest quintile in England for 2017.^13^

Following a review of the cases of MRSA, improvements to processes and practices have been implemented. This includes routine screening of inpatients staying longer than 40 days.^14^

The reported indicators of AMR for our institution during this period were more variable. The rolling average proportion of gentamicin-resistant *E. coli* was in the middle quintile. The equivalent indicators for ciprofloxacin- and piperacillin/tazobactam-resistant *E. coli* were in the highest and second highest quintiles in England respectively.^13^ There were 17 cases of carbapenemase-producing Enterobacteriaceae in our institution in the financial year 2016–17. Comparative data for carbapenem resistance was not available at the time of writing. During this period our institution implemented national guidance for the identification and management of the most resistant microorganisms.^14^

In contrast with the hospital setting, the development of AMR after antibiotic treatment has been well described in primary care. A systemic review by Costelloe *et al.*^15^ found that individual patients prescribed antibiotics for respiratory or urinary tract infections developed AMR, with the effect greatest in the month immediately after treatment and persisting for up to 12 months.^15^ In light of the small numbers of resistant isolates in our study, such studies examining AMR at the level of the individual patient are of particular interest for the high-quality evidence linking antibiotic use to AMR.

Allowing for the many differences between community and ICU care, the residual effect holds the potential to influence a more tailored approach to antimicrobial therapy in the PICU. There is a case for more considered use of antibiotics generally, particularly for those children with multiple PICU admissions, exposed to multiple courses of antibiotics.

### Limitations

This study was a retrospective cohort review of patients with HCAIs. The limitations of this approach are that the methods of sampling, investigation and documentation in the electronic patient record were not designed for the purposes of the study. In a prospective study, knowledge of these criteria may increase the vigilance for diagnostic observations and investigations documented, resulting in fewer missed cases compared with a retrospective study. Retrospective cohort reviews are less useful for drawing associations between risk factors such as central venous catheters, compared with prospective studies.^16^

Our study was limited to positive cultures obtained from the bloodstream, endotracheal secretions or urine. The study did not include infections of other body sites nor viral or fungal infections.

The CDC definition of certain types of HCAI, for example urinary tract infection, permits a diagnosis to be made without an organism being cultured. In contrast, positive microbiology was a requirement in our study. This excluded cases that may have been considered as HCAI according to the CDC definition.

### Conclusions

The incidence of HCAI in our study (1.34%) is significantly lower than the lowest previously reported incidence of HCAIs, both in the paediatric intensive care setting and in the wider healthcare context. Critically ill children with HCAI were more likely to have comorbidities and an increased LOS. Among the HCAI identified, 30% were resistant Gram-negative organisms. The risk posed by HCAI and AMR is likely to increase with imprudent use of antimicrobials, the increasing number of children who have repeated admissions to inpatient care facilities and international travel from areas with high rates of AMR.

The challenges of HCAI and AMR may increasingly impact bed availability and other health resources. They demand a continuous formal programme of surveillance and rigorous infection control measures across all settings but particularly in areas of high risk such as the PICU.^12^
